# Advances in ovarian cancer radiomics: a bibliometric analysis from 2010 to 2024

**DOI:** 10.3389/fonc.2024.1456932

**Published:** 2024-10-01

**Authors:** Wang Lan, Jiang Hong, Tan Huayun

**Affiliations:** Department of Obstetrics, Weifang People's Hospital, Shandong Second Medical University, Weifang, China

**Keywords:** bibliometric, ovarian cancer, ovary neoplasm, radiomics, imaging biomarkers

## Abstract

**Objective:**

Ovarian cancer, a leading cause of death among gynecological malignancies, often eludes early detection, leading to diagnoses at advanced stages. The objective of this bibliometric analysis is to map the landscape of ovarian cancer radiomics research from 2010 to 2024, emphasizing its growth, global contributions, and the impact of emerging technologies on early diagnosis and treatment strategies.

**Methods:**

A comprehensive search was conducted using the Web of Science Core Collection (WoSCC), focusing on publications related to radiomics and ovarian cancer within the specified period. Analytical tools such as VOSviewer and CiteSpace were employed to visualize trends, collaborations, and key contributions, while the R programming environment offered further statistical insights.

**Results:**

From the initial dataset, 149 articles were selected, showing a significant increase in research output, especially in the years 2021-2023. The analysis revealed a dominant contribution from China, with significant inputs from England. Major institutional contributors included the University of Cambridge and GE Healthcare. ‘Frontiers in Oncology’ emerged as a crucial journal in the field, according to Bradford’s Law. Keyword analysis highlighted the focus on advanced imaging techniques and machine learning.

**Conclusions:**

The steady growth in ovarian cancer radiomics research reflects its critical role in advancing diagnostic and prognostic methodologies, underscoring the potential of radiomics in the shift towards personalized medicine. Despite some methodological challenges, the field’s dynamic evolution suggests a promising future for radiomics in enhancing the accuracy of ovarian cancer diagnosis and treatment, contributing to improved patient care and outcomes.

## Introduction

Ovarian cancer is a highly lethal malignancy in the female reproductive system. Its high mortality rate is primarily due to the high proportion of patients diagnosed at an advanced stage. Although medical technology has advanced in recent years, including developments in surgical and chemotherapy treatments, early diagnosis and prognosis assessment are crucial to improving patient survival rates (1). Against this backdrop, radiomics is an emerging interdisciplinary field that uses high-throughput technologies to analyze large datasets from medical images (2–4). It offers new perspectives and methods for the early diagnosis and treatment of ovarian cancer (5–7).

Radiomics, through quantitative analysis of imaging biomarkers, can non-invasively reveal the microscopic structure and biological characteristics of tumors. This provides a basis for personalized treatment of ovarian cancer patients. Additionally, omics technologies can be used for early disease diagnosis, treatment outcome prediction, and patient prognosis (8, 9). However, the integration of radiomics with other omics data, such as genomics and proteomics, is still underdeveloped, limiting a comprehensive understanding of tumor biology and its applications in personalized medicine.

As research in the field continues to grow, integrating and analyzing information from a vast amount of literature to identify research hotspots and trends poses a significant challenge for researchers. Bibliometrics provides a powerful tool for understanding the knowledge structure, research dynamics, and future directions of a field by quantitatively analyzing various characteristics of a literature collection. In the interdisciplinary field of radiomics and ovarian cancer, bibliometrics can assist researchers in tracking technological progress and application trends, as well as revealing interconnections between different studies and potential research gaps. Despite the growing body of work, there is a notable lack of large-scale, multi-center studies that validate radiomic models across diverse populations, which is essential for ensuring their clinical reliability and applicability. Furthermore, the potential of radiomics in early-stage ovarian cancer detection remains underexplored, despite its critical importance in improving patient outcomes. This can guide future research directions.

## Methods

### Data source

We chose the Web of Science Core Collection (WOSCC) for its comprehensive coverage of scholarly publications across multiple disciplines, particularly in medical and scientific research. WOSCC is known for its detailed records of peer-reviewed articles and reviews, making it the preferred database for bibliometric analyses (10). The extensive metadata provided by WOSCC allows for in-depth exploration of citation patterns and research networks, offering insights that are often unparalleled compared to other databases. Furthermore, WOSCC’s ability to facilitate co-citation analysis is crucial for understanding literature relationships and identifying key trends in radiomics and ovarian cancer research (11).

### Search strategy

We employed a targeted search syntax designed to encompass a broad spectrum of terms related to radiomics, imaging biomarkers, and ovarian cancer. Specifically, the search terms used were:

TS = [(“Radiomics” OR “Imaging Biomarkers”) AND (“ovarian neoplasm*” OR “ovarian cancer*” OR “ovarian carcinoma” OR “ovary cancer*” OR “ovary tumor*” OR “ovary neoplasm*” OR “ovary carcinoma” OR “ovarian tumor*”)]

### Time frame

The temporal scope of our search spanned from January 1, 2010, to June 28, 2024, covering the most recent years. This period was deliberately chosen to encompass the development and evolution of radiomics and imaging biomarkers within ovarian cancer research, tracing its journey from emerging concepts to their current established state in the scientific community.

### Data selection

Two authors independently conducted a literature search in the Web of Science Core Collection (WOSCC) database using a predefined search string. This search was designed to capture a comprehensive dataset relevant to ovarian cancer radiomics. The search retrieved a total of 158 documents, including titles, article citations, keywords, author information, abstracts, publication countries or regions, and references, all of which were downloaded in TXT format for further analysis.

Following the initial search, we applied specific exclusion criteria to refine the dataset. Document types such as proceeding papers, meeting abstracts, editorial materials, book chapters, letters, corrections, notes, and non-English literature were excluded to ensure the analysis focused on peer-reviewed research articles and reviews. Specifically, 6 editorial materials and 3 meeting abstracts were excluded, resulting in 149 articles (comprising 115 research articles and 34 reviews) being retained for bibliometric analysis.

**Figure 1:**
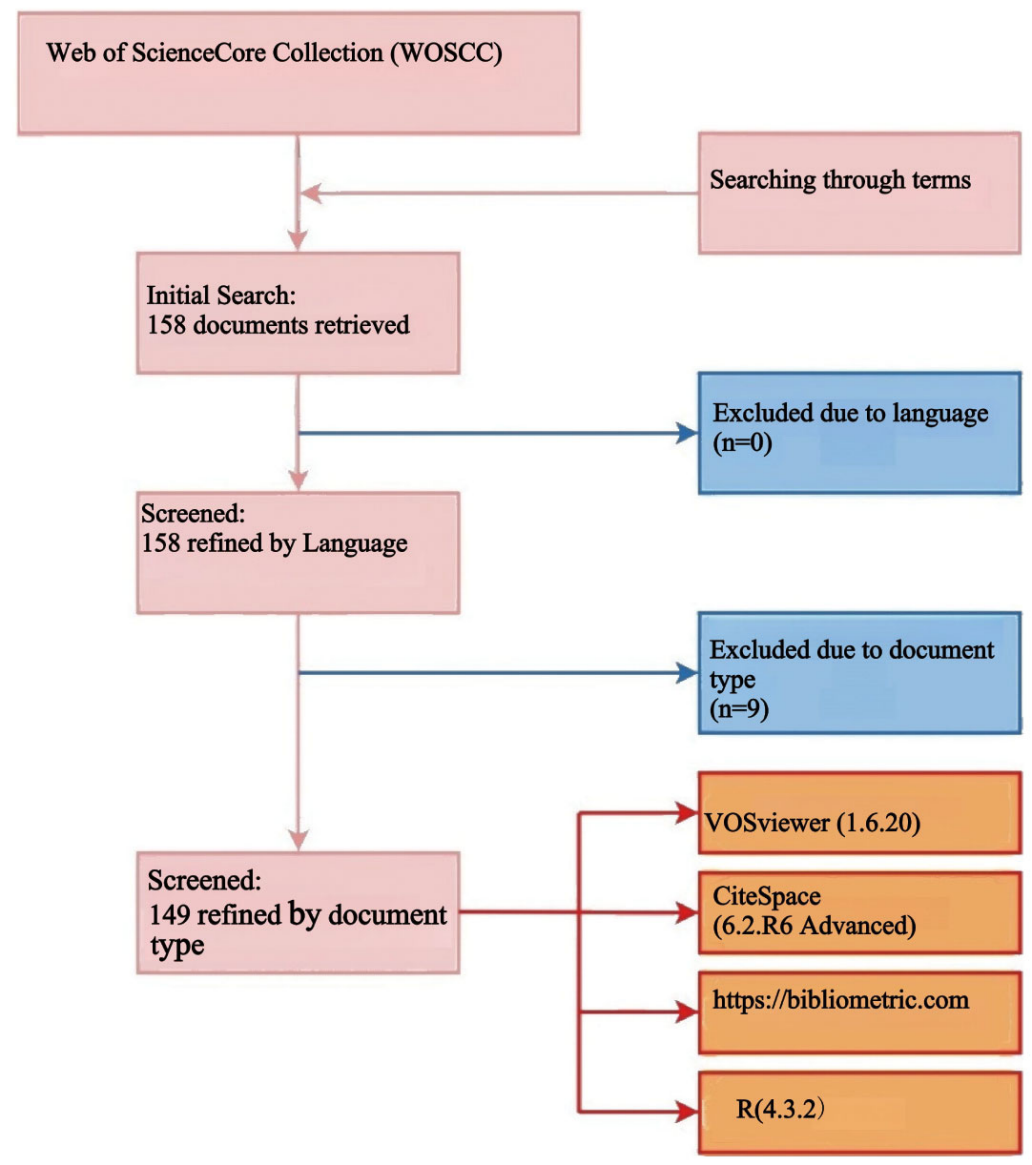
Literature screening and analysis workflow.

Any discrepancies between the two authors were resolved through discussion, ensuring consistency and accuracy. The entire literature selection process is visually represented in the flowchart. The entire literature selection process is visually represented in the flowchart (**Figure 1**).

### Bibliometrics and visualization analysis

We utilized several key software tools for our bibliometric analysis:

VOSviewer (version 1.6.20): VOSviewer is a powerful tool designed for creating and visualizing bibliometric networks, which include various types of relationships such as keyword co-occurrence networks and keyword hotspots. It is particularly well-suited for handling large datasets and producing clear, interpretable visualizations (12).

CiteSpace (version 6.2.R6 Advanced): Developed by Prof. Chaomei Chen, CiteSpace is widely used for detecting intellectual structures, research frontiers, and dynamics in scientific literature (13). It was used to analyze and visualize co-occurrence networks of countries and regions, universities and institutions, and authors; keyword citation bursts; dual maps of journals; timelines of keyword clusters; and cited reference bursts. Specific settings included time slicing from January 2010 to June 2024, with 1-year intervals. Default settings were used for text processing, node types, link strength, and selection criteria. Minimum spanning trees and pruning sliced networks were applied in keyword burst analysis, while pathfinder and pruning sliced networks were used for co-cited reference cluster analysis.

Microsoft Excel 2021 was used to visualize annual publication numbers.

R programming environment (version 4.3.2), in conjunction with online bibliometric analysis platforms (http://bibliometric.com/) and the bibliometrix package (version 4.0.0), was used to evaluate publication trends and identify core journals (14).

## Results

### Number of publications and distribution of publication years

**Figure 2:**
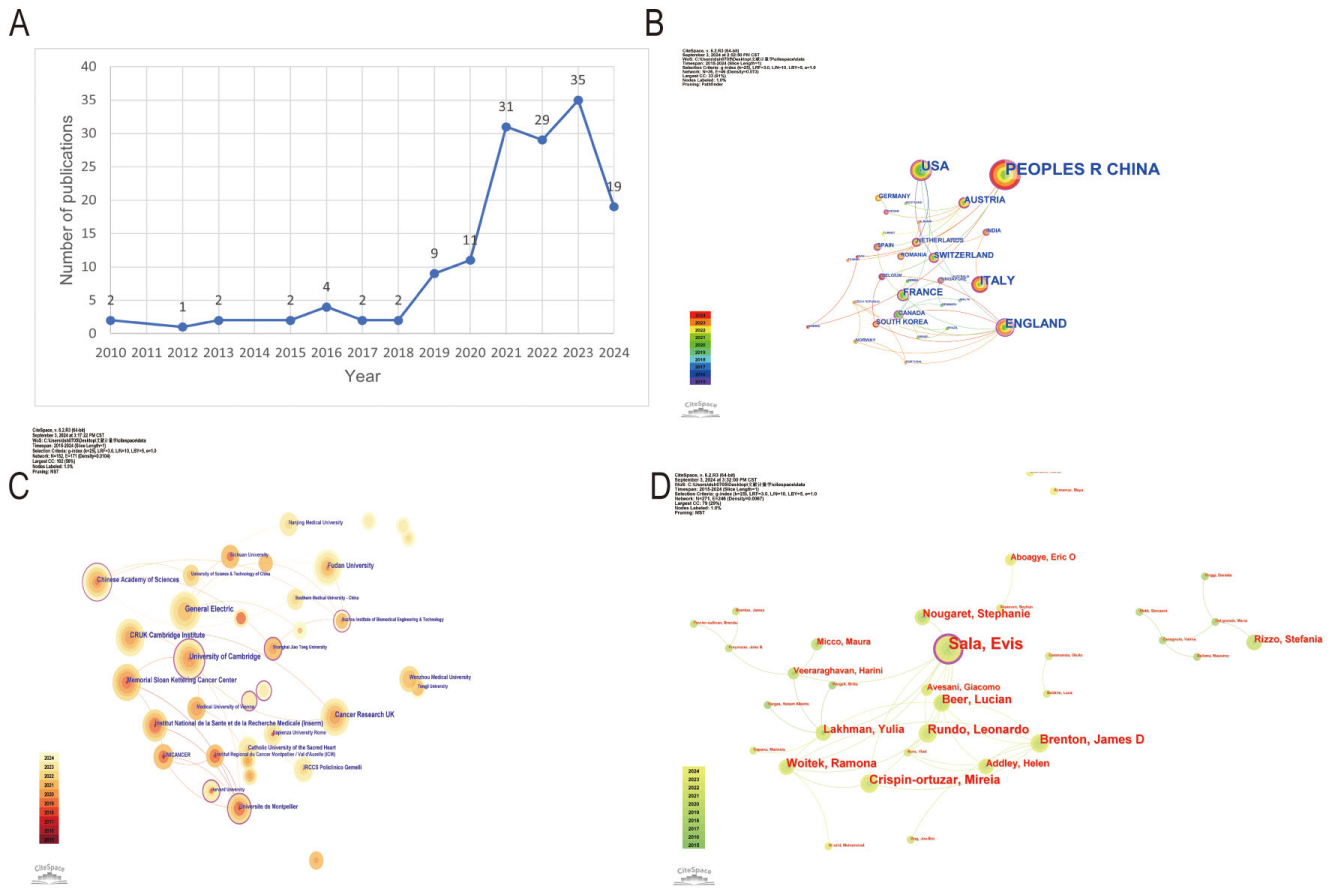
Overview of ovarian cancer radiomics research trends and collaborations. **(A)** Time trend distribution of publications. **(B)** Visualization of cooperation networks of countries or regions. **(C)** Visualization of cooperation networks among universities and institutions. **(D)** Visualization of cooperation networks among authors.

From 2010 to 2018, the annual number of publications remained stable, ranging between 1 and 4 per year. However, starting in 2019, there was a significant increase in publication output, reaching a new height in 2020 and peaking at 35 publications in 2022. In the first half of 2024 alone, 19 publications have already been recorded, indicating continued active research in this field (**Figure 2A**).

### Countries and regions

The People’s Republic of China, with the largest node representing 83 publications, leads among the 36 contributing countries and regions. England stands out with the most connections to other countries, the most prominent outer purple circle in the network, and the highest centrality score of 0.64, confirming its crucial role as the main hub for international research collaborations (**Figure 2B**).

### Analysis of universities and institutions

In a network of 337 universities and institutions from 36 countries and regions, the University of Cambridge has the most prominent node, indicating leading research contributions with 14 publications and notable centrality (centrality 0.09). Other key institutions include Memorial Sloan Kettering Cancer Center (12 publications, centrality 0.05), Fudan University (12 publications, centrality 0.03), and GE Healthcare (9 publications in 2021) (**Figure 2C**).

### Statistical analysis of authors

The statistical analysis of authors in the field of ovarian cancer radiomics reveals that Sala is the most influential contributor with 14 publications and a centrality of 0.09 in 2017. Other significant authors include Brenton, Crispin-Ortuzar, and Rundo, each with 7 publications and notable centrality scores (0.04 for Brenton and Crispin-Ortuzar, 0.02 for Rundo in 2020). Beer and Woitek each contributed 6 publications in 2020, with Woitek having a centrality of 0.05 (**Figure 2D**).

### Statistical analysis of journals

**Figure 3:**
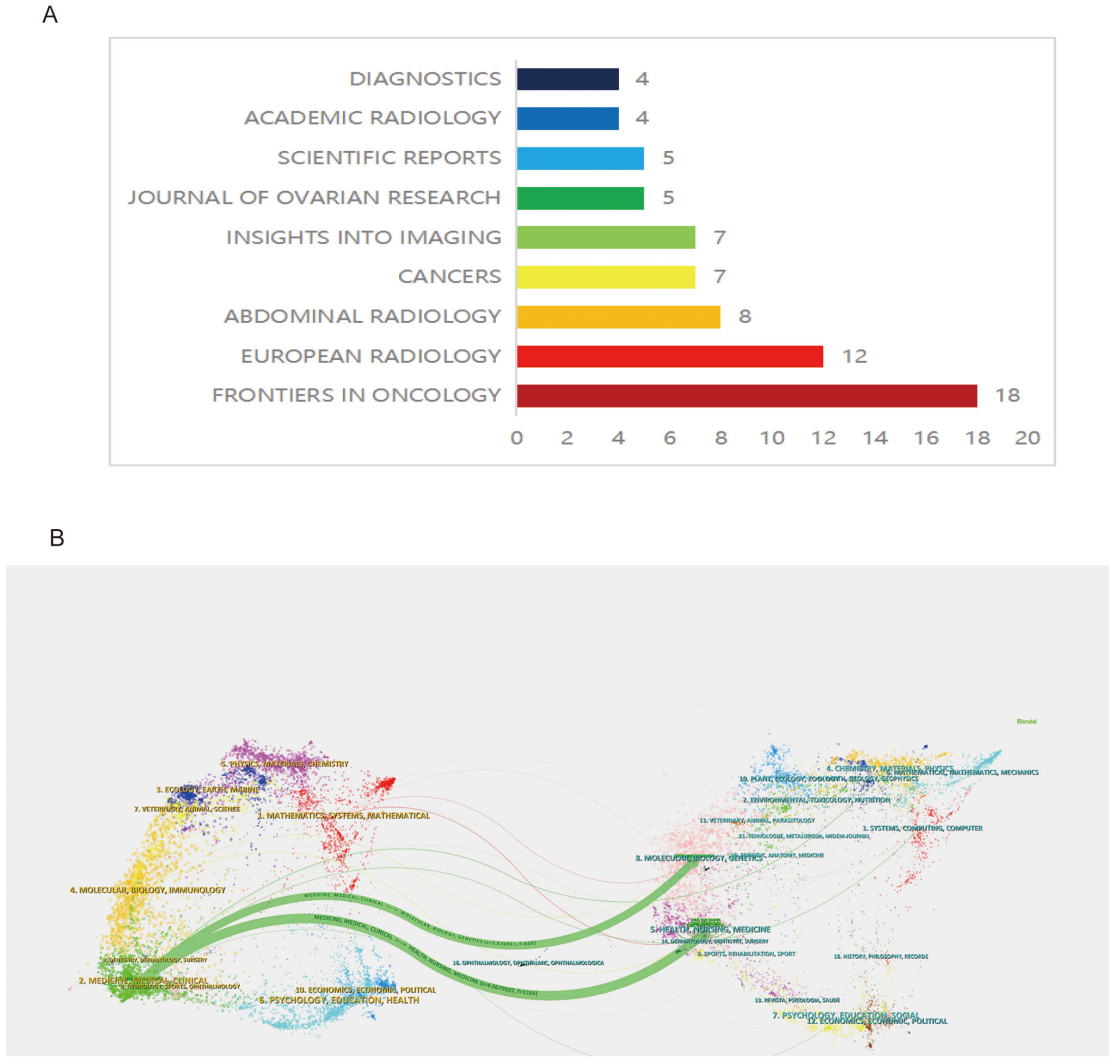
Analysis of journals **(A)** Brad’s Law Core Journals **(B)** Dual-map overlay of journals.

Within the specified field, out of 72 journals, 149 articles were published. According to Bradford’s Law, core journals include: Front. Oncol. (18 articles), Eur. Radiol. (12 articles), and Abdom. Radiol. (8 articles) (**Figure 3A**). Notably, Front. Oncol. stands out as the leading journal, publishing the highest number of articles, which underscores its pivotal role in disseminating significant research findings and advancements in ovarian cancer radiomics (**Figure 3A**).

### Analysis of the journal dual-map overlay

The analysis of the journal dual-map overlay offers a visual exploration of the scholarly landscape, mapping the citation relationships between diverse research fields. This approach delineates the multidisciplinary interactions and intellectual pathways that underpin scientific advancement, highlighting the dynamic exchange of knowledge between citing and cited journals across various domains. The dual-map overlay provides a nuanced visualization of scholarly communication, where journals on the left, predominantly from clinical research domains focusing on ovarian cancer, extensively reference and assimilate outcomes from molecular biology and biochemistry journals on the right. This citation pattern reveals a profound interdisciplinary exchange, illustrating how ovarian cancer clinical research is deeply entwined with, and significantly informed by, advancements in molecular biology and biochemistry (**Figure 3B**).

### Statistical analysis of keywords

**Table 1** highlights the frequency and centrality of key medical research terms in ovarian cancer radiomics. Prominent keywords include “ovarian cancer” (56 mentions, centrality 0.92), “computed tomography” (28 mentions, centrality 0.20), “cancer” (25 mentions, centrality 0.04), “machine learning” (22 mentions), and “magnetic resonance imaging” (21 mentions, centrality 0.23). These terms reflect significant advancements and focus areas in the field. Centrality measures how well a keyword connects different parts of the research network, indicating its importance in linking various studies.

**Table 1:** Top 10 keywords in the field of ovarian cancer radiomic.

Rank	Count	Centrality	Year	Keywords
1	56	0.92	2010	ovarian cancer
2	28	0.20	2019	computed tomography
3	25	0.04	2020	cancer
4	22	0.00	2021	machine learning
5	21	0.23	2019	magnetic resonance imaging
6	16	0.04	2021	prediction
7	16	0.04	2013	survival
8	15	0.00	2021	images
9	15	0.00	2017	features
10	13	0.21	2021	diagnosis

In the co-occurrence network visualization, larger nodes represent higher frequency of occurrences, while more connections indicate stronger centrality.

**Figure 4:**
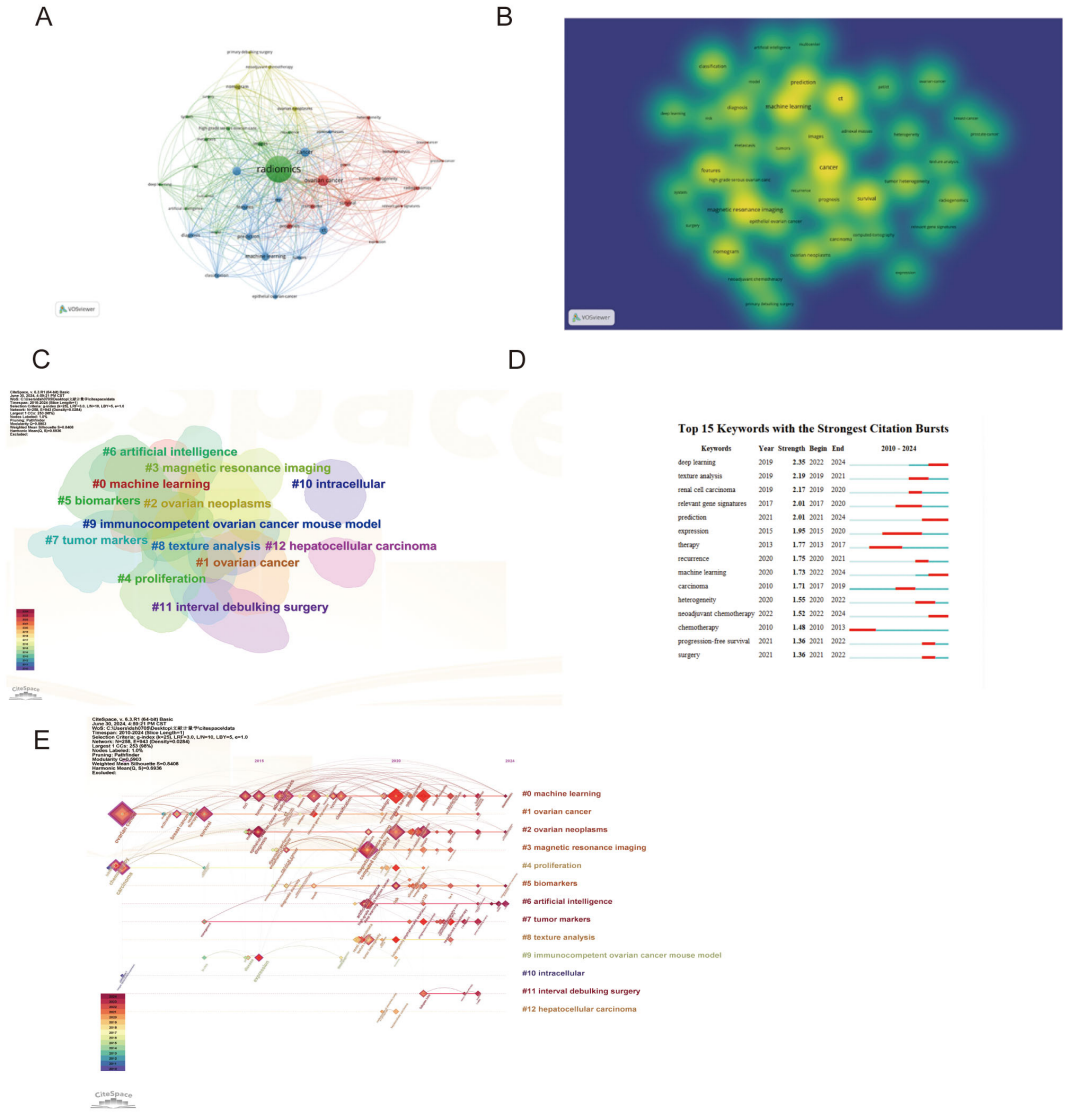
Analysis of keywords **(A)** Visualization of keywords co-occurrence **(B)** Visualization of keywords hotspot **(C)** Keywords clustering **(D)** Top 15 keywords with the strongest citation bursts **(E)** Clustering timelines of keywords.

**Figures 4A, B** displays the keyword co-occurrence graph and keyword hotspots with search terms removed, highlighting the key focus areas and technological advancements in the field.

### Analysis of keyword clusters and timelines

In keyword cluster diagrams and timelines, the number and size of keywords within a cluster indicate the volume of research and focus areas within that field. The smaller the number of groups (e.g., #0 “machine learning”), the more keywords within the cluster and the larger the cluster, indicating its prominence in the research field (**Figure 4C**). From the timeline diagram, keywords from the same cluster are placed on the same horizontal line, with more recent keywords towards the right, signifying that #0 machine learning remains the latest research hotspot in 2024 (**Figure 4E**).

### Analysis of keyword bursts

Recent trends show a significant increase in the application of advanced computational methods in ovarian cancer radiomics. Deep Learning (2019, Strength: 2.35) has seen a notable rise from 2022 to 2024, highlighting its growing importance in medical image analysis. Texture Analysis (2019, Strength: 2.19) was particularly prominent from 2019 to 2021, emphasizing its critical role in identifying tumor characteristics through imaging data. Additionally, Machine Learning (2020, Strength: 1.73) has gained substantial attention from 2022 to 2024, showcasing its expanding application in image analysis. These trends underscore the transformative impact of these technologies on precision medicine and personalized treatment strategies in ovarian cancer (**Figure 4D**).

### Analysis of cited references

**Figure 5:**
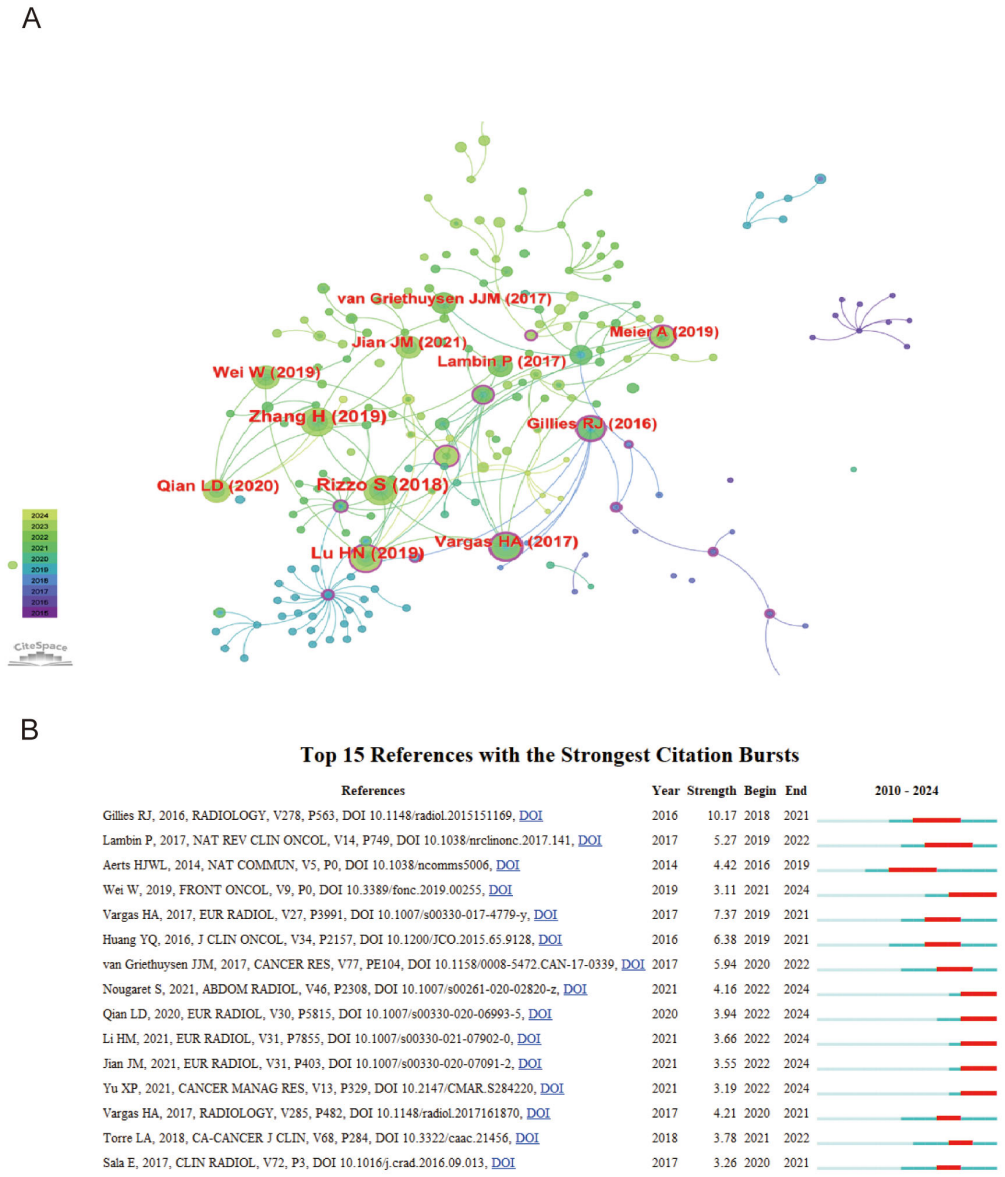
Analysis of cited references **(A)** Visualization of top 10 cited references **(B)** Top 15 cited references with the strongest citation bursts.

In the citation network, the article by Rizzo S (2018). stands out as a fundamental pillar in the study of radiomics, amassing a remarkable 43 citations, with 12 occurring in 2023 alone (15). This prominence is illustrated by the network’s largest node. Connections between papers, shown through lines, indicate co-citation relationships, suggesting thematic or contributory overlaps. Notably, the manuscript by Gillies RJ (2016). holds the highest centrality score of 0.37, distinguished by a purple outer circle, serving as an influential pivot within the network (16). This centrality underscores its critical role in shaping the research landscape (**Figure 5A**).

### Top 10 cited references

**Table 2** presents the top 10 most-cited references in the domain of radiomics for ovarian cancer, serving as landmark publications that have substantially influenced the field.

**Table 2:** Top 10 cited references in the field of ovarian cancer radiomics.

Rank	Citation Counts	References	Article Title
1	43	Rizzo S, 2018, EUR RADIOL (15)	Radiomics of high-grade serous ovarian cancer: association between quantitative CT features, residual tumour and disease progression within 12 months
2	39	Zhang H, 2019, EUR RADIOL (34)	Magnetic resonance imaging radiomics in categorizing ovarian masses and predicting clinical outcome: a preliminary study
3	37	Lu HN, 2019, NAT COMMUN (35)	A mathematical-descriptor of tumor-mesoscopic-structure from computed-tomography images annotates prognostic- and molecular-phenotypes of epithelial ovarian cancer
4	27	Vargas HA, 2017, EUR RADIOL (1)	A novel representation of inter-site tumour heterogeneity from pre-treatment computed tomography textures classifies ovarian cancers by clinical outcome
5	23	Qian LD, 2020, EUR RADIOL (39)	MR imaging of epithelial ovarian cancer: a combined model to predict histologic subtypes
6	23	Wei W, 2019, FRONT ONCOL (17)	A Computed Tomography-Based Radiomic Prognostic Marker of Advanced High-Grade Serous Ovarian Cancer Recurrence: A Multicenter Study
7	22	Jian JM, 2021, EUR RADIOL (40)	MR image-based radiomics to differentiate type I and type II epithelial ovarian cancers
8	22	Gillies RJ, 2016, RADIOLOGY (16)	Radiomics: Images Are More than Pictures, They Are Data
9	21	Lambin P, 2017, NAT REV CLIN ONCOL (41)	Radiomics: the bridge between medical imaging and personalized medicine
10	20	van Griethuysen JJM, 2017, CANCER RES (42)	Computational Radiomics System to Decode the Radiographic Phenotype

### Analysis of cited references burst

The analysis of top 15 cited references with the strongest citation bursts reveals key works that have significantly influenced ovarian cancer radiomics (**Figure 5B**). Notably, Gillies RJ (2016, Radiology) introduced the foundational concept of radiomics, transforming images into high-dimensional data for enhanced decision support (Strength: 10.17, 2018-2021) (16). A key study by Wei W (2019, Front Oncol) discussed the applications of radiomics in oncology, highlighting a computed tomography-based radiomic prognostic marker of advanced high-grade serous ovarian cancer (HGSOC) recurrence (Strength: 3.11, 2021-2024). This study emphasized that a radiomic signature and nomogram can be low-cost, non-invasive tools for predicting the risk of postoperative recurrence in advanced HGSOC, potentially enabling individualized patient evaluation (17).

## Discussion

The steady increase in publication volume from 2010 to 2023, with a notable surge in recent years, indicates growing recognition and exploration within the field of ovarian cancer radiomics. This upward trend, supported by early data from 2024, suggests ongoing interest driven by emerging technologies and methodologies. Recent key publications underscore this trend. Liu et al. (2024) (18) developed an ultrasound image-based nomogram combining clinical, radiomics, and deep transfer learning features for classifying ovarian masses according to O-RADS. Yu et al. (2024) (19) introduced a radiomics nomogram for differentiating early-stage serous borderline ovarian tumors from serous malignant ovarian tumors. Fu et al. (2024) (20) proposed multitask prediction models for serous ovarian cancer using preoperative CT image assessments based on radiomics. These advancements reflect the dynamic nature of ovarian cancer radiomics, driven by innovative technologies and collaborative research. The continuous rise in publications signifies the field’s growth and its promising future in transforming ovarian cancer diagnosis and treatment (18–20).

Geographically, the dominant contributions from China, followed by significant inputs from England, highlight global engagement, where diverse perspectives and expertise converge to advance the field. This international collaboration is mirrored in the institutional data, with entities like the University of Cambridge, General Electric, and Fudan University leading in publications, suggesting a strong network of research excellence and cross-institutional partnerships. The pivotal roles of authors like Evis Sala underscore the influence of individual researchers in shaping the domain’s landscape through significant contributions and collaborations.

Bradford’s Law illustrates the principle that a small number of journals are responsible for publishing the majority of important research. Among these, Frontiers in Oncology stands out with 18 publications, highlighting its substantial contribution to the field of oncology research (4, 5, 18, 19, 21–33). This demonstrates the journal’s central role in disseminating new knowledge and fostering progress in the field.

The foundational work by Rizzo et al. (2018) laid the groundwork for a paradigm shift in predicting the progression of high-grade serous ovarian cancer (15). This study underscored the potential of CT radiomics in forming personalized treatment plans and highlighted the critical role of integrating imaging biomarkers into clinical workflows, offering a nuanced and predictive approach to cancer care. Building on this groundwork, the 2019 study by Zhang et al. enhanced the diagnostic accuracy for ovarian cancer using MRI radiomics to classify ovarian masses (34). This research was pivotal in showcasing the precision MRI radiomics can offer in differentiating between ovarian mass types, thus enabling more accurate and timely treatment decisions.

Additionally, the innovative 2019 research by Lu et al. established a novel connection between radiomic features from imaging data and the molecular biology of ovarian cancer (35). By introducing a non-invasive method to predict the molecular and prognostic phenotypes of ovarian cancer, this study opened new paths for understanding the disease’s heterogeneity.

The article by Vargas HA et al., published in Radiology in 2015 with a moderate citation strength of 2.56 and a peak impact from 2017 to 2020, found significant associations between specific computed tomographic (CT) imaging features, particularly mesenteric infiltration and diffuse peritoneal involvement, with Classification of Ovarian Cancer (CLOVAR) subtypes and survival outcomes in patients with high-grade serous ovarian cancer (HGSOC) (36). In 2016, Gillies RJ published an article in Radiology, firmly establishing that in the realm of radiomics, images are not merely pictures but data (16). This seminal paper, which achieved a burst strength of 6.97 and recorded the highest centrality score of 0.37, meticulously introduced the concept of radiomics as the transformation of images into higher-dimensional data for subsequent mining to enhance decision support. Highlighting the inception of radiomics in oncology research with its potential applicability across all diseases, this landmark publication has had a profound impact on the field from 2018 to 2021, emphasizing its critical role in advancing medical imaging analysis and its integration into broader disease diagnosis and management strategies.

The 2021 publication by Li HM in European Radiology marks a pivotal advancement in radiomics, especially highlighting its capability to refine diagnostic and prognostic methodologies via sophisticated image analysis (37). Echoing the transformative work of Gillies RJ in 2016, Li’s research underscores the dynamic evolution of radiomics within the sphere of medical imaging. Significantly, this study showcases the utility of MRI-based radiomic nomograms for non-invasively predicting residual tumor presence in patients with advanced HGSOC, indicating a major leap forward in the application of non-invasive techniques for managing complex conditions. With a burst strength of 2.26, Li’s work not only enhances the precision of medical diagnostics but also marks a historic transition towards the integration of radiomics across medical fields. This progress represents a significant stride towards personalized medicine, emphasizing the growing relevance of radiomics in driving the future direction of research and clinical practices aimed at tackling complex diseases.

Yu XP’s 2021 study in Cancer Management and Research notably emphasizes the transformative role of radiomics within oncology, signaling a significant shift towards more sophisticated diagnostic and management strategies in cancer care (38). With a notable impact underscored by a burst strength of 2.18, the research highlights the pivotal use of radiomics, especially the application of features derived from Multidetector Computed Tomography (MDCT), in distinguishing between Serous Borderline Ovarian Tumors (SBOTs) and Serous Malignant Ovarian Tumors (SMOTs).

Collectively, these seminal studies have significantly propelled the field of ovarian cancer radiomics forward, setting new benchmarks for incorporating imaging and computational analysis in ovarian cancer management. Their contributions have enhanced the precision and efficacy of diagnostic and prognostic methods, aligning with the overarching aim of offering personalized, patient-centric treatment strategies. As we continue to unravel the complexities of ovarian cancer, the methodologies and insights provided by these researchers will undoubtedly continue to guide research directions and clinical practices, marking critical milestones in the continuous pursuit of precision medicine in oncology.

The use of specific databases and analytical tools, such as CiteSpace, highlights the strengths and limitations of current research methodologies in the field of ovarian cancer radiomics. The Web of WoSCC database is especially useful for co-citation analysis, identifying important publications that have influenced the field. Such analyses are crucial for understanding the development of research trends, pivotal theories, and technologies that have defined the progress of ovarian cancer radiomics. However, this reliance also introduces potential limitations. The requirement for WoSCC may restrict the inclusion of certain relevant publications not indexed within this database, potentially omitting valuable insights and data. Furthermore, the use of tools like CiteSpace can be complex and may result in variable outcomes with each import, posing challenges for researchers. Nevertheless, these variations generally do not diminish the overall research findings and conclusions.

These factors emphasize the need for reliable, high-quality data sources and robust, user-friendly analytical frameworks. The creation and implementation of these resources are crucial for advancing the field, allowing for a comprehensive and precise mapping of research trends, collaborations, and seminal works. Additionally, improving the accessibility and usability of analytical tools can democratize research efforts, enabling a broader range of researchers to contribute to the ongoing exploration and innovation in ovarian cancer radiomics.

## Conclusions

The bibliometric analysis of ovarian cancer radiomics research from 2010 to 2024 reveals a robust and expanding field, characterized by a significant increase in research output, particularly in recent years. This growth reflects the field’s potential to revolutionize early diagnosis, prognosis, and treatment of ovarian cancer through advanced imaging analytics. The global engagement in this research, led by countries like China and institutions such as General Electric and the University of Cambridge, highlights the collaborative efforts that are driving these advancements. Influential researchers like Evis Sala and leading journals such as Frontiers in Oncology play pivotal roles in disseminating key findings, shaping the trajectory of the field.

However, despite these advancements, several critical research gaps remain. The absence of large-scale, multi-center validation studies is a significant barrier to the broader clinical application of radiomics, limiting the generalizability and reliability of findings across diverse populations. Additionally, the underutilization of radiomics in early-stage ovarian cancer represents a missed opportunity to improve early detection, which is vital for enhancing patient survival rates. Furthermore, the limited integration of radiomics with other omics fields, such as genomics and proteomics, restricts the potential for developing more comprehensive and personalized treatment strategies.

Moving forward, addressing these research gaps is crucial for realizing the full potential of radiomics in ovarian cancer management. Future research should prioritize the development of large-scale, multi-center studies to validate radiomic models, explore the application of radiomics in early-stage ovarian cancer, and integrate radiomic data with other omics technologies. By focusing on these areas, the field can advance toward more precise, reliable, and personalized approaches to ovarian cancer diagnosis and treatment, ultimately contributing to better patient outcomes and the advancement of precision medicine in oncology.

## Data Availability

The original contributions presented in the study are included in the article/supplementary material. Further inquiries can be directed to the corresponding author.
